# The antitumor effect induced by an IL-2 ‘no-alpha’ mutein depends on changes in the CD8^+^ T lymphocyte/Treg cell balance

**DOI:** 10.3389/fimmu.2022.974188

**Published:** 2022-08-17

**Authors:** Tania Carmenate, Galia Montalvo, Sum Lai Lozada, Yaretnis Rodriguez, Yaquelin Ortiz, Claudia Díaz, Janet Avellanet, Juhee Kim, Charles D. Surh, Luis Graça, Kalet León

**Affiliations:** ^1^ Immune Regulation Department, Centro de Inmunología Molecular, Havana, Cuba; ^2^ Division of Integrative Biosciences and Biotechnology, Pohang University of Science 12 and Technology (POSTECH), Pohang, South Korea; ^3^ Instituto de Medicina Molecular, Faculdade de Medici na da Universidade de Lisboa, Lisbon, Portugal

**Keywords:** IL-2 mutein, treg, cancer therapy, CD8^+^ T cells, TME

## Abstract

High doses of interleukin-2 (IL-2) have been used for the treatment of melanoma and renal cell carcinoma, but this therapy has limited efficacy, with a ~15% response rate. Remarkably, 7%–9% of patients achieve complete or long-lasting responses. Many patients treated with IL-2 experienced an expansion of regulatory T cells (Tregs), specifically the expansion of ICOS^+^ highly suppressive Tregs, which correlate with worse clinical outcomes. This partial efficacy together with the high toxicity associated with the therapy has limited the use of IL-2-based therapy. Taking into account the understanding of IL-2 structure, signaling, and *in vivo* functions, some efforts to improve the cytokine properties are currently under study. In previous work, we described an IL-2 mutein with higher antitumor activity and less toxicity than wtIL-2. Mutein was *in silico* designed for losing the binding capacity to CD25 and for preferential stimulation of effector cells CD8^+^ and NK cells but not Tregs. Mutein induces a higher anti-metastatic effect than wtIL-2, but the extent of the *in vivo* antitumor activity was still unexplored. In this work, it is shown that mutein induces a strong antitumor effect on four primary tumor models, being effective even in those models where wtIL-2 does not work. Furthermore, mutein can change the *in vivo* balance between Tregs and T CD8^+^ memory/activated cells toward immune activation, in both healthy and tumor-bearing mice. This change reaches the tumor microenvironment and seems to be the major explanation for mutein efficacy *in vivo*.

## Introduction

IL-2 is a cytokine with a pivotal role in the control of immune system homeostasis, acting on both effector and regulatory lymphocytes (1). The IL-2 binds to the multimeric IL-2 receptor (IL-2R) expressed on different lymphocyte subsets and induces lymphocyte survival, proliferation, and activation. The heterotrimeric IL-2R is composed of three subunits: IL-2Rα (CD25), IL-2R β (CD122), and IL-2Rγc (CD132). The IL-2Rβ and IL-2Rγc chains are responsible for signaling and together form the dimeric intermediate affinity receptor (KD ~ 1 nM) highly expressed on memory phenotype (MP) CD8^+^CD44^hi^ and NK cells (2). The α-chain (CD25) is expressed constitutively on CD4^+^Foxp3^+^Tregs (3) and transiently on activated CD4^+^ and CD8^+^ T cells (4) and together with IL-2Rβand IL-2Rγc forms the high-affinity IL-2R (KD ~ 1 pM) (5, 6).

Based on its immune-stimulatory activity, IL-2 has been used in the treatment of advanced melanoma and renal cell carcinoma, but its high toxicity precludes its extended use (7). Nevertheless, it is notable that 15%–30% of treated patients experienced clinical improvements including long-lasting responses (8). However, some patients became unresponsive to the therapy, probably associated with the expansion of the highly suppressive ICOS^+^Tregs (9, 10). As IL-2-based therapy has not reached the expectancy, improving the IL-2 therapeutic index is still an active and important topic. Among other examples are the immunocytokine format for directing IL-2 to the tumor cells (11, 12), IL-2 fusion with the IgG-Fc region to increase the molecule half-life (13), or increasing the half-life and changing molecular properties with site-directed pegylation that has been assayed (14). Moreover, the design and evaluation of different muteins seem to be reasonable to increase therapy efficacy (15, 16). One of the ideas is to change the IL-2 distribution among different lymphocyte populations, based on the differential expression of IL-2Rαβγc or IL-2Rβγc. We previously described a mutein, named no-alpha, with a severe decrease in affinity to CD25 (16). No-alpha mutein behaves as an agonist with higher antitumor activity but lower toxicity than wtIL-2. In the present work, we extended the demonstration of the antitumor capacity of no-alpha mutein to four primary tumor models and survival induction in two spontaneous metastases models. Mutein was originally designed to induce preferentially CD8^+^ and NK *in vivo* expansion and activation, but this issue remained to be fully demonstrated *in vivo*. Here we demonstrate that mutein can change the CD8^+^MP/Treg balance *in vivo* in both healthy and tumor-bearing mice. This change toward immunity reaches the tumor microenvironment, and it is mainly due to the specific increment in CD8^+^ T-cell proliferation without any important change in Treg accumulation or activation.

## Methods

### Mice

Seven- to eight-week-old female C57BL/6 and BALB/c mice were obtained from The National Center for Laboratory Animal Breeding (Havana, Cuba), Animal Resources Centre, IBS Pohang Institute of Basic Science (Pohang, Korea), or Instituto Gulbenkian de Ciencia (Oeiras, Portugal). Food and water were administered *ad libitum*. The experiments were performed according to the International Guidelines for the Care and Use of Laboratory Animals, using standardized procedures of the three institutes.

### Production and purification of hIL-2 no-alpha mutein from insoluble material

The hIL-2 no-alpha mutein was produced in *Escherichia coli*, and the isolation of inclusion bodies and purification of the protein were performed as previously described (16). Briefly, the frozen pellets were suspended in 10 mM of Tris and 1 mM of EDTA (pH 8) (TE) and sonicated using an ultrasonic cell disrupter (IKA, Wilmington, NC, USA). The insoluble material was harvested by centrifugation (18,000 ×*g*) and washed successively with 4 M of urea-TE and 1% Triton X-100–TE using an Ultra Turrax T8 homogenizer. For further purification, aGE AKTA explorer system was used; the protein was extracted with 6 M of guanidinium hydrochloride–TE at 0.1 g/ml (wet weight), and renaturation was carried out by dialysis. For further purification, protein was applied to a reverse-phase C4 column (Vydac). In this final chromatography, the recombinant polypeptides were purified using an H_2_O–acetonitrile–trifluoroacetic acid system, with a linear gradient (30%–85% of acetonitrile) and 0.6 ml/min flow. Finally, the proteins were dialyzed against 10 mM of acetate (pH 4), filtered through 0.2-µm filters, and stored at 4°C. The molecule was tested for low endotoxin levels, and the biological activity was evaluated using the CTLL-2 cell proliferation assay.

### *In vivo* effect on T-cell populations

Healthy mice were treated with wtIL-2 (20,000 or 40,000 international units (IU)) or no-alpha mutein (200 or 400 IU) for 6 days twice a day. After the treatments, the spleens were harvested, and the cell suspension was obtained, stained, and analyzed by flow cytometry. For assessing BrdU incorporation, mice were fed with BrdU in the water 3 days before sacrifice. The BrdU staining was performed with the specific kit from BD Biosciences (San Jose, CA, USA) following the fabricant instructions.

### Antibodies and flow cytometry

All fluorochrome-conjugated mAbs used for flow cytometry measurement were from Thermo Fisher (Waltham, MA, USA) unless otherwise stated: fluorescein isothiocyanate (FITC) or BV-conjugated anti-CD3 (145-2C11), PE or APC-conjugated anti-CD4 (L3T4), PB or FITC-conjugated anti-Foxp3 (NRRF-30), PE-Cy7-conjugated anti-CD25(3C7), APC-Cy7-conjugated anti-CD8 (eBio H35-17.2), BV605 or APC anti-CD44 (IM7), PE-conjugated anti-CD122(5H4), and APC-conjugated anti-CD45(30-F11). Intracellular Foxp3 staining sets were purchased from eBioscience (San Diego, CA, USA). Live/dead fixable near IR dye from Thermo Fisher Scientific was used for dead cell discrimination. Samples were measured using a flow cytometer Gallios (Beckman Coulter, Brea, CA, USA) or LSR Fortessa (Becton Dickinson, Franklin Lakes, NJ, USA) and analyzed using Kaluza software or FlowJo software (TreeStar, Inc., San Carlos, CA, USA).

### Tumor challenges and treatments

Mouse transplantable tumor cell lines used melanoma MB16, colon CT26, mammary tumor 4T1, lung carcinoma 3LL-D122, and lung carcinoma TC1 cells maintained in Dulbecco’s modified Eagle medium (DMEM) F12 (GIBCO, Grand Island, NY, USA) supplemented with 10% heat-inactivated fetal bovine serum, 2 mM of l-glutamine, 50 U/ml of penicillin, 50 μg/ml of streptomycin. All cells were maintained at 37°C under a humidified 5% CO_2_ atmosphere. Tumor cells were harvested using trypsin/EDTA and resuspended in phosphate-buffered saline (PBS) for *in vivo* experiments. All the tumor cells were inoculated in the right flank s.c., except the 4T1 cells that were inoculated in the mammary gland and the 3LL-D122 cells that were inoculated in the footpad. For the 3LL-D122 spontaneous metastasis model, the primary tumors were eliminated by surgery when they reached a diameter of 9 mm. Treatments consisted of two cycles of five daily i.p. injections of PBS, wtIL-2 (30,000 IU), or no-alpha mutein (300 IU) twice a day, and the cycle was repeated after one resting week. Tumor size was assessed using a microcaliper every 3 days, and the tumor volume was calculated using the following formula: (width^2^ × length)/2. For the spontaneous metastases models with 3LL-D122 and 4T1 cells, the mouse survival was also monitored periodically for 80 days. For tumor-infiltrating lymphocyte evaluation, tumors were mechanically dissociated. First, the tumors were cut into small pieces and then meshed through a 70-µm cell strainer using a syringe plunger. The obtained cell suspensions were centrifuged, stained, and analyzed by flow cytometry.

### Data and statistical analysis

For statistical analysis, the Graph Pad Prism 4.0 software was used. For comparison of the CD8/Treg ratios, and BrdU incorporation levels in healthy mice parametric ANOVA followed by Tukey’s multiple comparison test were applied. In the antitumor assays, a two-way ANOVA followed by Bonferroni’s test was applied for comparison of the tumor curves; for survival analysis, the log-rank test was used. In the case of CD8/Treg ratio in tumor-bearing mice, the Kruskal–Wallis test followed by Dunn’s test for multiple comparisons was used.

## Results

### IL-2 no-alpha mutein is able to modify the CD8^+^MP/Treg balance in healthy mice

**Figure 1:**
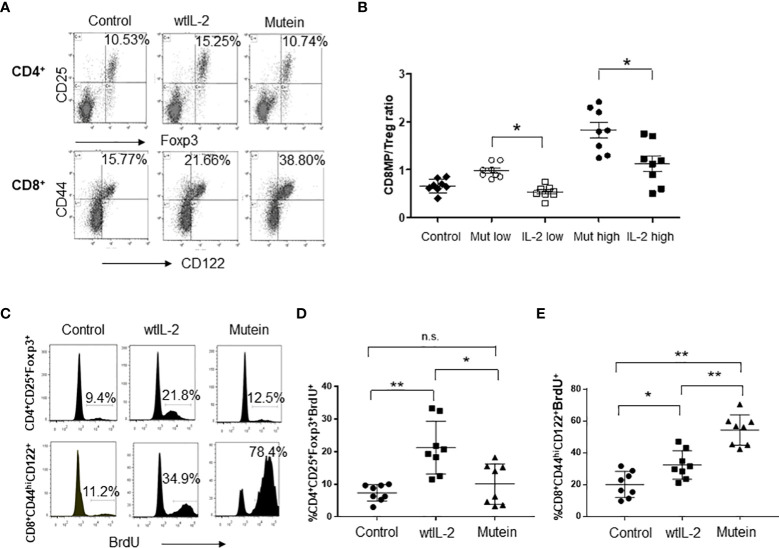
No-alpha mutein induces accumulation and proliferation of CD8^+^MP cells but not Tregs. Mice were treated i.p. with 20,000 IU of wtIL-2 or 200 IU of mutein during a week, and the percentages and numbers of CD8^+^MP and Tregs on the spleens of treated mice were determined. **(A)** Representative dot plots. **(B)** CD8^+^MP/Treg cell ratio measured on mice treated with two different doses of wtIL-2 and no-alpha mutein: low doses, 20,000 IU of wtIL-2 or 200 IU of mutein; high doses 40,000 IU of wtIL-2 or 400 IU of mutein. The experiment was performed three times; representative data are presented, n = 8 per treatment, p ˂ 0.05, Bonferroni test. For BrdU incorporation, mice were treated with 20,000 IU of wtIL-2 or 200 IU of mutein and fed with BrdU in the water for the last 3 days of treatments. **(C)** Representative histograms of BrdU incorporation. **(D, E)** Cumulative data from two independent experiments, n = 8, Tukey’s test was used for multiple comparisons (p < 0.001 **; p < 0.05 *). n.s., non significant.

The IL-2-derived no-alpha mutein was previously characterized by its capacity to stimulate *in vitro* cells with different forms of IL-2R. To evaluate the effect of the IL-2 no-alpha mutein on CD8^+^MP and Treg balance *in vivo*, B6 mice were treated with both molecules, and the accumulation and proliferation of both populations on the spleens of treated mice were measured. Mice were treated with 20,000 IU of wtIL-2 or 200 IU of mutein, and the selected doses expressed as protein mass correspond to 20 µg of wtIL-2 and 20 µg of no-alpha mutein and are equivalent for cells expressing the IL-2Rβγc, for which the two molecules induce similar levels of proliferation *in vitro* (16). **Figure 1A** shows representative results obtained in the experiments. As expected, the mice treated with mutein showed higher percentages of CD8^+^MP cells, reaching 38.8%, and the wtIL-2 induced a lower accumulation level of 15.25% still above the value of 10.7% in the control group. On the contrary, the wtIL-2 was the only molecule able to increase the percentage of Tregs among the CD4^+^ T lymphocytes, reaching approximately 15% in comparison with the typical 10% observed in the control group and the group treated with mutein. To test the overall impact on the balance effector/regulatory cell, mice were treated with two different doses of each molecule: 20,000 or 40,000 IU for wtIL-2 and 200 or 400 IU for mutein. Total CD8^+^CD44^hi^CD122^+^ T cells and CD4^+^CD25^+^Foxp3^+^ cells on the spleens were quantified. The mice treated with mutein showed a higher CD8^+^MP/Treg ratio than the mice treated with wtIL-2 in both dose levels. The results show that regardless of the dose, mutein is always able to expand CD8^+^MP T cells preferentially (**Figure 1B**). To define if the observed accumulation was related to an increment in proliferation, the incorporation of BrdU by the cells in treated mice was measured. **Figure 1C** shows representative histograms, and **Figures 1D, E** show cumulative data from two independent experiments. Mutein induced an outstanding increment on BrdU^+^ CD8^+^MP cells, in a range from 40% to almost 80% of CD8^+^MP cells in the S phase of the cell cycle. The wtIL-2, on the contrary, induced a higher accumulation of Tregs, with an increment in the percentages of BrdU positive Tregs, in a range from 11% to 32% being statistically different from the average level observed in the control group 7.4% (**Figure 1D**).

### IL-2 no-alpha mutein reduces primary tumor growth and is able to modify the CD8/Treg balance in tumor-bearing mice

**Figure 2:**
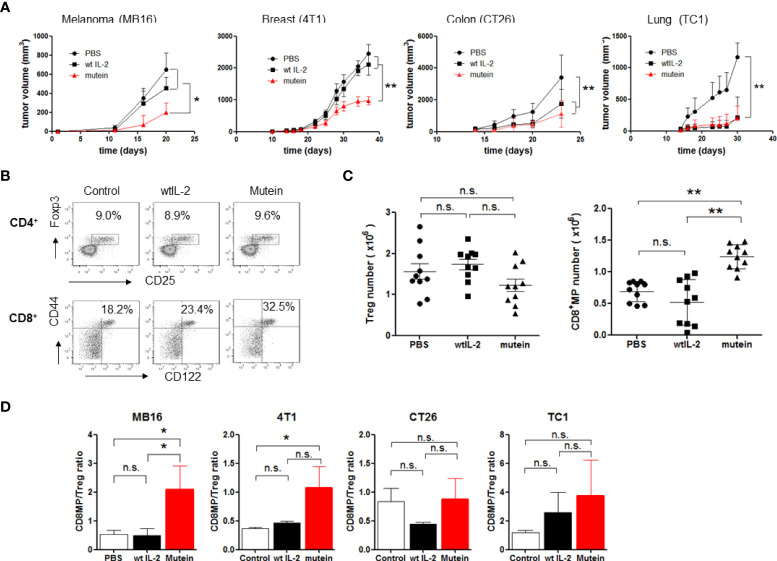
No-alpha mutein induces a potent antitumor effect in four different tumor models and is able to change CD8^+^MP/Treg balance in tumor-bearing mice in the spleens. Mice were inoculated with tumor cells and received 300 IU of mutein or 30,000 IU wtIL-2, twice a day, for 2 weeks separated by a resting week. MB16, CT26, and TC-1 cells were inoculated s.c. on the right flank; 4T1 cells were inoculated on mammary glands. **(A)** Tumor growth curves. **(B)** Representative dot plots from spleen of MB16-bearing mice. **(C)** CD8^+^MP and Treg number from spleens of MB16-bearing mice. **(D)** CD8^+^MP/Treg ratios on spleens of mice inoculated with the different tumor cell lines. Cumulative data from three experiments, n = 9 per treatment. Statistical differences in tumor growth were determined by two-way ANOVA test, using GraphPad software (p < 0.001 **; p < 0.05 *). n.s., non significant.

It was previously demonstrated that no-alpha mutein induces an important anti-metastatic effect in the MB16 and 3LL-D122 experimental metastasis tumor models (16). Now we extended the study of the antitumor activity to other models with tumor cell lines from different origins. In the models for primary tumor growth studied (CT26, MB16, TC1, and 4T1), mutein was able to delay tumor growth (**Figure 2A**). The most important finding was that in two models (MB16 and 4T1) in which the wtIL-2 did not show any effect, mutein was highly effective in delaying the primary tumor growth, and the difference between mutein and the wtIL-2 was statistically significant for both tumor models (p < 0.05*, p < 0.001**).

Next, we evaluated the capacity of the IL-2 mutein to change the balance of CD8^+^MP/Tregs in tumor-bearing mice. Although different tumors induce different suppressive mechanisms (17), we have previously studied that all the tumors used in the present work expand Tregs on mouse spleens above the normal levels, even when other suppressive populations can also be expanded. We focused on the balance of CD8/Tregs because of the well-known function of IL-2 stimulating these populations. Furthermore, the results confirm our hypothesis about the mechanism of action of mutein.

We evaluated the accumulation of CD8^+^MP and Tregs on the spleens of tumor-bearing mice treated with IL-2 or mutein on day 21 after tumor implant. First, we selected the MB16 tumor model because it is one of the tumor models where mutein showed a higher antitumor effect than the wtIL-2. **Figure 2B** shows representative results of CD8^+^MP and Treg measurement on melanoma-bearing mice treated with the different molecules, and **Figure 2C** shows cumulative data of cell number. There were no statistical differences in Tregs number among the groups treated with wtIL-2, mutein, or PBS as control. However, the percentage and number of CD8^+^MP cells increased notably in the mice treated with no-alpha mutein, reaching 32% of CD8^+^ cells and more than 1 × 10^6^ cells, while the mice treated with the wtIL-2 only showed a discreet increase in the percentages reaching 23% of CD8^+^MP cells close to the value of the control group (18%); nevertheless, the number of activated CD8^+^ cells was similar for the group treated with the wtIL-2 and the control group. The increment on CD8^+^MP cells but not in Tregs results in a change in the CD8^+^MP/Treg balance toward immune activation (**Figure 2D**). This change on CD8^+^MP/Treg balance was also observed for the 4T1 model and, to a less extent, not statistically different for CT26 and TC1 models. Interestingly, the two models where mutein induced the highest effect on primary tumor growth (MB16 and 4T1) were those with statistical differences in the CD8^+^MP/Tregs ratio between the groups treated with mutein and the wtIL-2.

### IL-2 no-alpha mutein increases the survival of tumor-bearing mice and changes the CD8^+^/Treg balance in the tumor microenvironment

**Figure 3:**
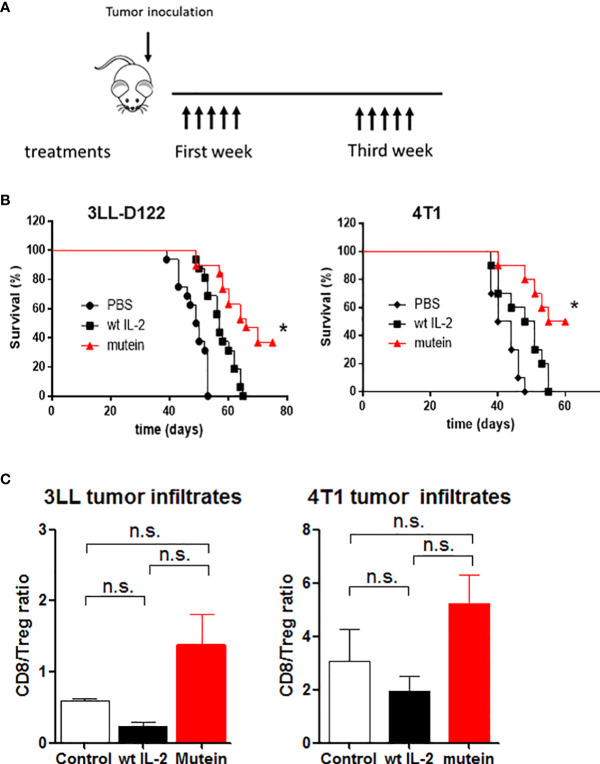
No-alpha mutein induces higher survival than wtIL-2 and induces changes in the tumor microenvironment. Mice were inoculated with 4T1 cells in the mammary gland or with 3LL-D122 on the foot pad; in the case of 3LL-D122, the primary tumors were eliminated by surgery when the control reached 9 mm; mice were treated with 30,000 IU wtIL-2 and 300 IU of no-alpha mutein or phosphate-buffered saline (PBS), and survival was observed. **(A)** Treatment schedule. **(B)** 3LL-D122 and 4T1 survival curves from spontaneous metastasis development. Survival curves were compared by log-rank test. **(C)** For tumor infiltrate measurement, mice were sacrificed when tumors reached 5–6 mm diameter and analyzed by flow cytometry. The graph represents the ratio between percentages of CD8^+^T lymphocytes and Treg referred to as total CD45^+^ cells. n.s., non significant. *p< 0.05.

No-alpha mutein effect on survival of tumor-bearing mice was also tested. The experimental tumor models 4T1 and 3LL-D122 were selected due to their capacity for inducing spontaneous metastases. Mice were treated with 30,000 IU of wtIL-2 or 300 IU of mutein; these doses are equivalent in the ability to stimulate effector cells, both CD8^+^ activated and NK cells expressing the dimeric IL-2 receptor (βγc). Similar to the traditional treatment used in humans, the molecules were administered twice a day for 5 days, and the same treatments were repeated a week apart (**Figure 3A**). In both cases, the mice treated with mutein showed a higher survival ratio than the mice treated with the wtIL-2 (p < 0.05), and both groups showed better survival than the control group treated with PBS (**Figure 3B**).

To study if the effect on immune balance reaches the tumor microenvironment, mice inoculated with 4T1 and 3LLD-122 cells were treated as described above. After the primary tumors were 6–7 mm in diameter, the mice were sacrificed, and the tumors were collected and analyzed by flow cytometry. TILs were evaluated as percentages of CD8^+^ or CD4^+^CD25^+^Foxp3^+^ T lymphocytes from the total number of CD45^+^ cells, and the ratio between percentages is shown (**Figure 3C**). The tumors treated with mutein showed a higher level of CD8^+^T lymphocytes than the tumors treated with the wtIL-2, inducing a desired increment in the balance CD8^+^/Tregs in the tumor microenvironment.

## Discussion

IL-2 is a pleiotropic cytokine with a central role in the control of the immune response (1). Due to its effect on both effector and regulatory T cells, IL-2 has been evaluated as a therapeutic tool in a wide range of diseases related to cancer and autoimmunity (8, 18). After several years of using high-dose IL-2 therapy for cancer treatment, many approaches are under study aiming to improve the therapeutic index of the cytokine.

We have previously described the design and evaluation of an IL-2 mutein with a reduced binding capacity to the alpha chain of the IL-2R, named ‘no-alpha mutein’; mutein shows a higher anti-metastatic effect and less toxicity than wtIL-2, opening the opportunity to a better IL-2-based tumor therapy (16). Now the evaluation of the antitumor effect of mutein was expanded to other tumor cell lines and experimental settings. Mutein showed a strong effect in delaying primary tumor growth and inducing a better survival ratio in the models of spontaneous metastases induced by 4T1 and 3LL-D122 cell lines. Moreover, the effect on activated CD8^+^MP cells vs Tregs balance in the spleens was measured in healthy and tumor-bearing mice. Mutein was able to increase CD8^+^MP/Treg ratio in both settings, validating the hypothesis that it is enough to eliminate the interaction of the IL-2 molecule with the receptor alpha chain to achieve a preferential expansion of IL-2Rβγc-expressing cells. The effect of mutein was also measured in the tumor microenvironment of 4T1 and 3LL-D12 models, and a change in the CD8^+^/Treg balance was found.

The selection of the appropriate dose to compare the *in vivo* effect was an important choice. Usually, the IL-2 dose used in humans corresponds to 600,000 IU/kg, and it is considered a high dose treatment; in most tumor experiments in mice, an equivalent dose of 30,000 IU is used, corresponding with 1–3 μg of wtIL-2 per dose. For mutein, as well as for the wtIL-2, the biological activity is determined in the cell proliferation assay for the CTLL-2 cell line expressing the IL-2Rαβγc. As mutein only binds to the dimeric receptor, more molecules are needed to achieve the same level of CTLL-2 proliferation. We considered the 100-fold difference in the affinity of the IL-2Rαβγc vs the IL-2Rβγc and chose 300 IU as the equivalent dose for mutein; this dose corresponds with 10 to 30 μg of mutein depending on the specific activity of the production batch. Mutein has shown lower toxicity than wtIL-2; consequently, it is possible to use 30 μg or higher doses without inducing severe toxic effects on treated mice.

When the selected doses were evaluated *in vivo*, the activated CD8^+^CD44^hi^CD122^+^T cells accumulated to higher levels in the healthy mice treated with mutein than in the mice treated with wtIL-2. To study the CD8^+^MP/Treg ratio in healthy mice, two dose levels were evaluated; mutein showed a significant change toward immune activation rather than suppressive condition. Taking into account the CD8^+^ preferential expansion demonstrated *in vivo*, we can speculate that when different lymphocyte populations are present and compete for the cytokine, mutein is captured preferentially by the CD8^+^ cells, which express a high level of the dimeric βγc IL-2R form.

In this work, we explored how general could be the advantage of no-alpha mutein over wtIL-2 and used several tumor models regardless of whether Treg expansion is the main suppressive mechanism or not. We demonstrated that in the models with higher Treg expansion, such as MB16 and 4T1, the differences in the antitumor activity between mutein and the wtIL-2 were higher as well as the changes induced on CD8^+^MP/Treg ratio. Consequently, mutein should be advantageous in the treatment of those cancer diseases where the Treg expansion is relevant; in these settings, we expect better efficacy and lower percentages of non-responder patients due to lower levels of ICOS^+^Treg proliferation. A phase I clinical trial evaluating mutein safety is already in progress.

Expansion of Tregs in lymphoid organs and the tumor microenvironment is one of the tumor mechanisms to escape from immune surveillance (19). In humans, Treg increment is related to worse outcomes in many tumor diseases, and the expansion of ICOS-positive Tregs is related to IL-2 therapy unresponsiveness (9). Several examples are attempting to target Tregs in order to achieve significant antitumor responses; antibodies against CTLA-4, GITR, CXCR4, ICOS, and CD25 have been used for this purpose (20–24), although the restricted expression of the target molecules on Tregs can be sometimes questioned. Vargas *et al.* provided an elegant demonstration of specific CD25^+^ expression on Tregs in both mice and human tumor infiltrates. They demonstrated that increasing the CD25 mAb PC-61 affinity to activating FcγRs was sufficient to specifically deplete Tregs in the tumor microenvironment and to improve the antitumor effect of mAb alone or in combination with PD-1 mAb (25). Another outstanding finding was the induction of systemic antitumor response after intratumor Treg depletion with a mAb targeting CTLA-4 molecule, underscoring the relevance of Tregs as immunosuppressive mechanisms in tumor settings (22). Also, there is the work from Cheung *et al.*, which improved the old concept of the ONTAK molecule, with the production of a second-generation IL-2 diphtheria toxin fusion protein with higher activity and better formulation than the previous one, and the demonstration of Treg depletion in spleens and LN together with a potent antitumor activity (26).

In the present work, not Treg depletion but changes in the balance of CD8/Tregs were achieved, treating the mice with no-alpha mutein. The goal was to abolish Treg expansion mediated by IL-2 while conserving the immunostimulatory properties of the cytokine-activating effector cells. Recently a similar idea was accomplished through a different approach (14). Charych *et al.* developed a modified IL-2, with a site-directed PEG attachment on the alpha surface of the molecule; the modified IL-2 (NKTR-214) also increased the CD8^+^/Tregs ratio and has a higher half-life, which could be an advantage for treatment schedule but also a problem considering the toxic effects associated with the IL-2 molecule. NKTR-214 shows a potent antitumor effect both alone and in combination with a PD-1 mAb. Also, the first phase I clinical trial was conducted by assaying safety and dose regimen for the NKTR-214 molecule; moderate responses were observed together with several adverse events associated with the treatment. Levin *et al.* also described an IL-2 mutein with agonistic properties (15); H-9 has an increased affinity for the β chain of IL-2R, and although it conserves the capacity to induce Treg proliferation, also induced an outstanding effect on CD8^+^ cells and induces higher antitumor activity than wtIL-2.

In conclusion, our results extend previous studies from our group and other laboratories showing that IL-2 muteins can lead to an antitumor effect *in vivo* by expanding effector CD8^+^ T cells while restricting the expansion of Treg cells. The benefit of this approach for cancer treatment will now be assessed under clinical trials that have been already initiated.

## Data availability statement

The raw data supporting the conclusions of this article will be made available by the authors, without undue reservation.

## Ethics statement

This study was reviewed and approved by Ethic Committees for laboratory animal care and use at the CIM, Havana; Postech, Korea and at the IMM, Lisbon.

## Author contributions

TC, GM, YO designed and performed *in vivo* antitumor experiments. TC, JK, JA, YO, design and performed *in vivo* experiments for lymphocytes changes. CD, JA, TC, performed tumor microenvironment measurements. SLL, YR purified wtIL-2 and IL-2 mutein. TC wrote the document, CHDS, LG and KL design experiments, generated ideas and discuss the experiment designs and results. All authors contributed to the article and approved the submitted version.

## Funding

The research was supported by Cuban Academy of Science and by the Cuban government. The authors also acknowledge the financial help received from Professor Charles D’Surh, and Professor Luis Graça.

## Conflict of interest

The authors declare that the research was conducted in the absence of any commercial or financial relationships that could be construed as a potential conflict of interest.

## Publisher’s note

All claims expressed in this article are solely those of the authors and do not necessarily represent those of their affiliated organizations, or those of the publisher, the editors and the reviewers. Any product that may be evaluated in this article, or claim that may be made by its manufacturer, is not guaranteed or endorsed by the publisher.

## Abbreviations

Foxp3, forkhead box P3 transcription factor; γ_c_, common γ-chain; IL-2R, IL-2 receptor; IU, international units; Tregs, regulatory T cells; wtIL-2, wild-type human IL-2.
